# Ascl3 transcription factor marks a distinct progenitor lineage for non-neuronal support cells in the olfactory epithelium

**DOI:** 10.1038/srep38199

**Published:** 2016-12-02

**Authors:** Pei-Lun Weng, Mridula Vinjamuri, Catherine E. Ovitt

**Affiliations:** 1 Department of Pathology and Laboratory Medicine, University of Rochester School of Medicine and Dentistry, Rochester, New York, 14642, USA; 2 Center for Oral Biology and Department of Biomedical Genetics, University of Rochester School of Medicine and Dentistry, Rochester, New York, 14642, USA

## Abstract

The olfactory epithelium (OE) is composed of olfactory sensory neurons (OSNs), sustentacular supporting cells, and several types of non-neuronal cells. Stem and progenitor cells are located basally, and are the source of all cell types needed to maintain OE homeostasis. Here, we report that Ascl3, a basic helix-loop-helix transcription factor, is expressed in the developing OE. Lineage tracing experiments demonstrate that the non-neuronal microvillar cells and Bowman’s glands are exclusively derived from Ascl3^+^ progenitor cells in the OE during development. Following chemically-induced injury, Ascl3 expression is activated in a subset of horizontal basal cells (HBCs), which repopulate all microvillar cells and Bowman’s glands during OE regeneration. After ablation of Ascl3-expressing cells, the OE can regenerate, but lacks the non-neuronal microvillar and Bowman’s gland support cells. These results demonstrate that Ascl3 marks progenitors that are lineage-committed strictly to microvillar cells and Bowman’s glands, and highlight the requirement for these cell types to support OE homeostasis.

The mammalian olfactory epithelium (OE) is a pseudostratified epithelium composed predominantly of olfactory sensory neurons (OSNs), which are generated in the basal region and extend apically to the nasal cavity. They are supported by an apical layer of glial-like sustentacular cells^1^,^2^. Scattered throughout the OE are the non-neuronal microvillar cells and Bowman’s glands. Bowman’s glands consist of clustered acinar cells located under the OE in the lamina propria, linked to ducts that span the epithelium to transport mucus to the apical surface^3^. At least three types of microvillar cells have been described in the OE^4^. Two types, distinguished by different morphologies, express the transient receptor potential channel M5 (Trpm5)^5^. The third type is characterized by expression of phospholipase C β2 (PLC β2), and type 3 IP_3_ receptor (IP3R3), both involved in calcium-mediated signal transduction, and of CD73^6^,^7^. The latter microvillar cell type has been identified as the primary source of neuropeptide Y (NPY) in the OE, which binds specific receptors to stimulate proliferation of basal progenitor cells and neurogenesis^8^,^9^. Knockout of NPY, or its receptor, results in reduced stem cell proliferation and decreased production of OSNs^9^,^10^. Numerous lines of evidence have indicated that the microvillar cells play an important role in OE homeostasis^9^,^11^,^12^,^13^.

The OE undergoes constant turnover, which is fueled by basally located proliferative progenitors, and quiescent stem cells^14^,^15^,^16^. Under normal conditions, a heterogeneous population of active progenitors, known as globose basal cells (GBCs), expressing markers such as Lgr5, Ascl1, c-Kit or SEC8 generates the cell types to maintain the integrity of the OE^17^,^18^,^19^,^20^,^21^,^22^,^23^. In contrast, the multipotent horizontal basal cells (HBCs) are relatively quiescent, and are activated only after extensive lesioning of the OE, which removes both sustentacular cells and GBCs^14^. Re-activated HBCs can regenerate all cell types in the OE^14^,^24^.

Ascl genes, members of the achaete scute-like complex family, are basic helix-loop-helix transcription factors (bHLH), which are expressed in progenitor cells of various tissues at the time of cell type specification. In the OE, Ascl1 is found in a subset of GBCs, which give rise to OSNs and sustentacular cells^22^. A second family member, Ascl2, is a critical regulator of intestinal stem cell fate and follicular T-helper cell specification^25^,^26^. Ascl3, the least characterized member of the family, is a marker of progenitor cells in the salivary glands, and Ascl3-expressing precursor cells generate both duct and acinar cells *in vitro*^27^,^28^,^29^.

Here we demonstrate that Ascl3 is also expressed in the OE, and marks precursors of the non-neuronal microvillar cells and Bowman’s glands during development and regeneration. We report that Ascl3 expression is activated in progenitors during development, and in a subset of HBCs immediately after injury, which generate all microvillar cells and the Bowman’s glands. Using cell-specific ablation and injury-induced regeneration, we show that in the absence of these cell types, neurogenesis of OSNs is reduced but not blocked, which may be due to the decreased number of GBCs and increase in apoptotic cells. Our data provide new insight into the lineage of non-neuronal support cells, and their requirement for OE homeostasis.

## Results

### Precursors of non-neuronal support cells express Ascl3 during OE development

**Figure 1:**
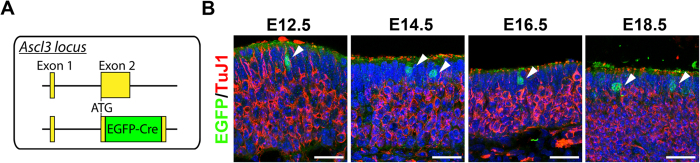
Ascl3 is expressed in the OE during embryonic development. **(A)** The Ascl3 gene locus includes 2 exons. In *Ascl3*^*EGFP-Cre*/+^ mice, the non-coding exon1 was maintained, and a fusion cassette encoding EGFP-Cre replaced the entire Ascl3 coding sequence in exon 2, through homologous recombination. **(B)** OE was isolated from *Ascl3*^*EGFP-Cre*/+^ mice at E12.5, E14.5, E16.5 and E18.5 of development. Immunohistochemistry was performed using antibodies to EGFP and TuJ1. Ascl3-EGFP^+^ cells (arrowheads) remain apically localized and are not colocalized with the marker for immature neurons, TuJ1, during embryonic development. Dotted line indicates basal lamina. Nuclei are stained by DAPI (blue). Scale bars: 25 μm.

To investigate the expression profile of Ascl3 during embryonic development of the OE, we used a previously generated mouse strain carrying a fusion cassette of EGFP and Cre recombinase knocked into the *Ascl3* gene locus, which replaced the entire Ascl3 coding sequence (Fig. 1A)^29^. In this strain, EGFP expression is driven by the endogenous *Ascl3* promoter. We observed EGFP as early as embryonic day 12.5 (E12.5) in the developing OE (Fig. 1B). EGFP-positive cells were detectable throughout embryonic development, at E14.5, E16.5 and E18.5, in cells localized at the apical region of the developing OE (Fig. 1B). There was no overlap observed between the EGFP-labeled cells and OSNs labeled with antibody to TuJ1.

**Figure 2:**
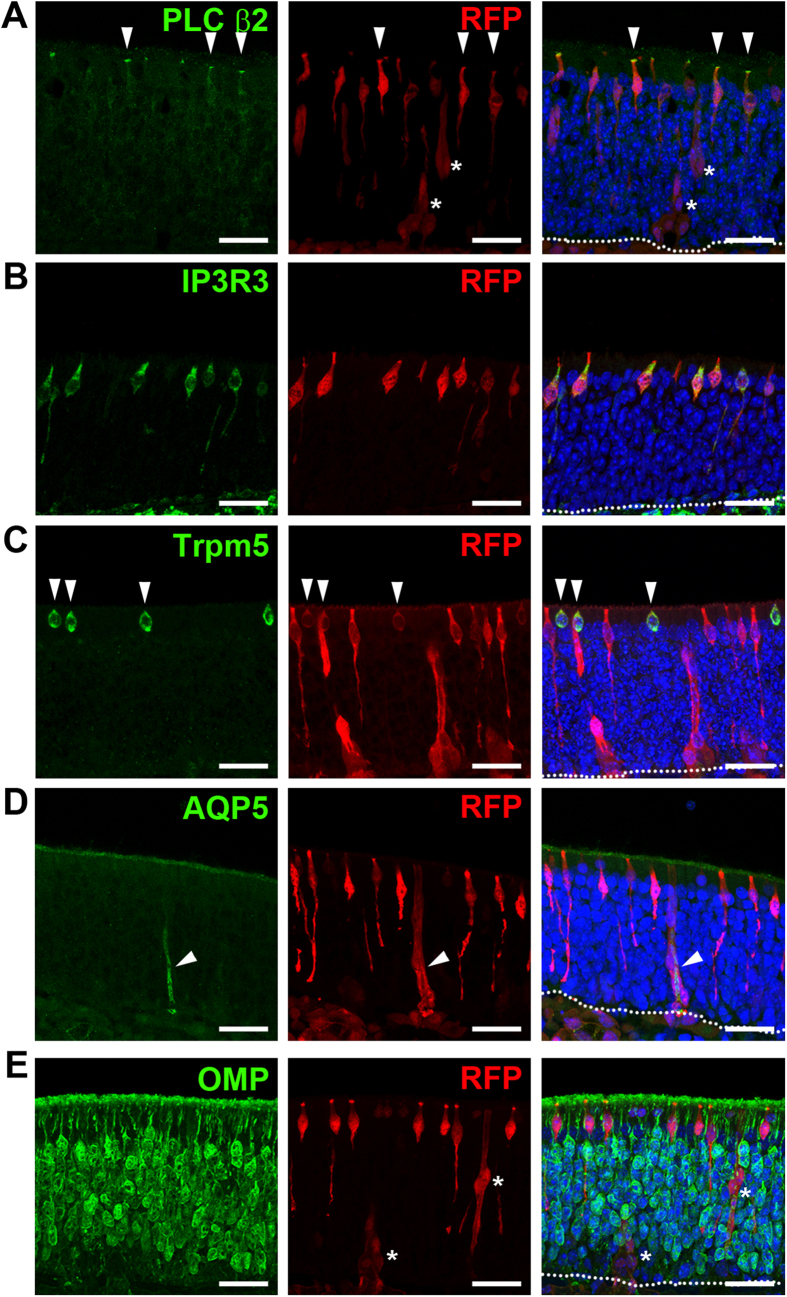
Ascl3-expressing cells are precursors of microvillar cells and Bowman’s glands. Immunohistochemistry was performed on OE isolated from *Ascl3*^*EGFP-Cre*/+^/*R26*^*tdTomato*/+^ mice (2 months), using antibodies to tdTomato (RFP) and (**A**) PLC β2, which marks the apical microvilli of microvillar cells, (**B**) IP3R3, (**C**) Trpm5, (**D**) AQP5 and (**E**) OMP. RFP expression colocalized with microvillar cell markers: PLC β2 (arrowheads), IP3R3 and Trpm5 (arrowheads) and Bowman’s glands markers: AQP5 (arrowheads). (**E**) No colocalization was detected between RFP and the mature OSN marker OMP. White asterisks mark Bowman’s gland duct cells. Dotted line indicates basal lamina. Nuclei are stained by DAPI (blue). Scale bars: 25 μm.

To follow the fate of the Ascl3-expressing cells found in the embryonic OE, we traced the lineage of these cells, using the *Ascl3*^*EGFP-Cre*^ strain crossed with the *R26*^*tdTomato*^ reporter. In *Ascl3*^*EGFP-Cre*/+^/*R26*^*tdTomato*/+^ mice, Ascl3-expressing cells, as well as their descendants, will be labeled with the red fluorescent protein reporter (RFP). RFP-positive cells were found in the mature OE, which display the morphology of apical microvillar cells. Their identity was confirmed by co-staining OE sections from adult *Ascl3*^*EGFP-Cre*/+^/*R26*^*tdTomato*/+^ mice (2 months) with antibodies to RFP, and to cell specific markers. RFP co-localized with the microvillar cell markers PLC β2, and IP_3_R3 (Fig. 2A,B). In addition, RFP also co-localized with cells expressing Trpm5, the cation channel specifically associated with a second type of microvillar cells (Fig. 2C). Morphology and cell-specific marker staining rule out that the apically localized RFP-positive cells are sustentacular cells. We conclude that the Ascl3 transcription factor is expressed by the lineage generating all types of microvillar cells.

In addition, RFP labeled structures co-localized with aquaporin 5 (AQP5), a marker for duct cells of Bowman’s glands that span the OE, as well as of basally located acinar cells in the lamina propria (Fig. 2D, arrowheads, Fig. S1A, arrows and arrowheads). In contrast, there was no co-localization between RFP-labeled cells and mature OSNs labeled with OMP (Fig. 2E). Moreover, we found no evidence of RFP-labeling of sustentacular cells, GBCs or HBCs under non-injury conditions. Lineage tracing experiment using the *Ascl3*^*EGFP-Cre*^ strain crossed with the *R26*^*confetti*^ reporter gave results consistent with those described above. All labeled cells exhibited the morphology of microvillar cells or Bowman’s glands (Fig. S1B; YFP and RFP channels shown), but other cell types were not labeled. Taken together, we conclude that Ascl3 is activated in progenitors, which exclusively generate the secretory microvillar cells and Bowman’s glands.

### Ascl3 expression is maintained in the NPY^+^ microvillar cells in the adult olfactory epithelium

**Figure 3:**
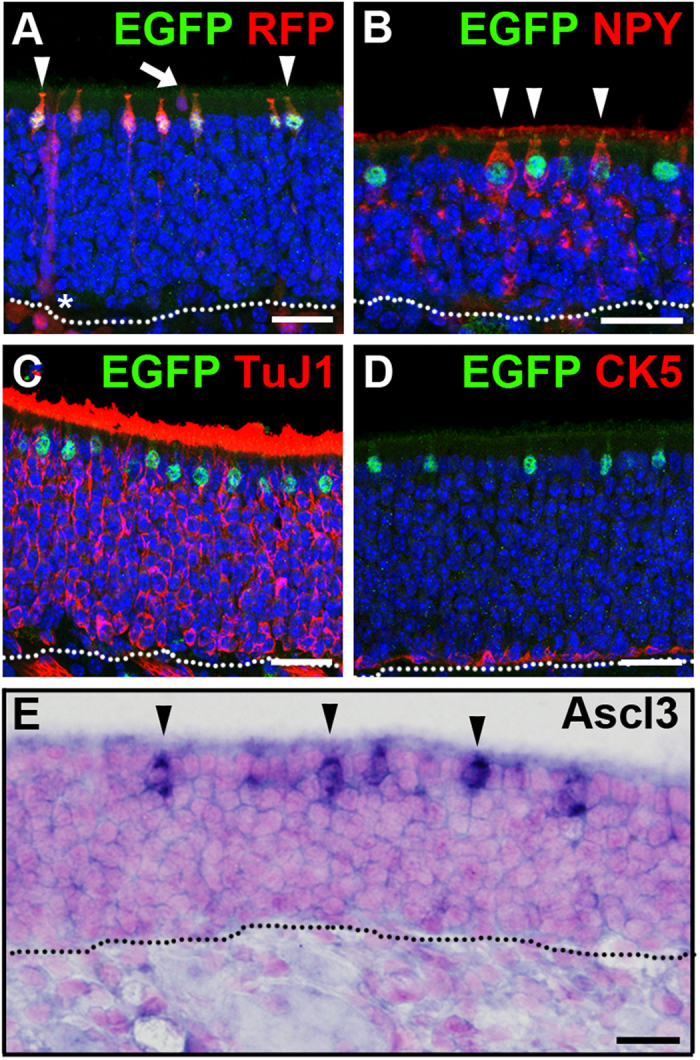
Ascl3 is expressed in the adult olfactory epithelium. (**A**) Immunohistochemistry was performed on sections of OE isolated from *Ascl3*^*EGFP-Cre*/+^/*R26*^*tdTomato*/+^ mice (2 months) using antibodies to EGFP and RFP. (**B**–**D**) Immunohistochemistry was performed on sections of OE isolated from *Ascl3*^*EGFP-Cre*/+^ mice (2 months), using antibodies to EGFP and (**B**) NPY, (**C**) TuJ1, (**D**) CK5. Arrowheads indicate co-localization of EGFP and NPY. (**E**) *In situ* hybridization for Ascl3 mRNA in the OE of 2-month old mice (arrowheads). Dotted line indicates basal lamina. Nuclei are stained by DAPI (blue). Scale bars: (**A**–**D**), 25 μm. (**E**), 20 μm.

Further examination showed that a subset of RFP-positive cells in the adult OE co-localizes with antibody to EGFP (Fig. 3A). The identity of these cells was determined using antibodies to cell type specific markers. Co-localization of antibodies to EGFP and NPY showed that mature NPY^+^ microvillar cells express Ascl3 (Fig. 3B). However, there was no EGFP expression detected in the Bowman’s glands or Trpm5+ microvillar cells (Fig. 3A). No overlap of EGFP with either OSNs or with cytokeratin 5 (CK5), a marker of the basally located HBCs was found (Fig. 3C,D). *In situ* hybridization on adult OE confirmed that Ascl3-driven EGFP expression recapitulates the endogenous distribution of Ascl3 mRNA (Fig. 3E). Thus, the expression of Ascl3 is maintained in the apical NPY^+^ microvillar cells of adult OE, but not in the Bowman’s glands or Trpm5+ microvillar cells. We conclude that Ascl3 expression in progenitors of the latter cell types must occur only transiently during fetal development. Furthermore, in contrast to Ascl1^21^, Ascl3 expression was not detected in the basal region harboring the stem and progenitor cells of the adult OE (Fig. 3B–E).

### Knockout of Ascl3 does not alter microvillar or Bowman’s gland cell differentiation

To investigate a possible role for the Ascl3 transcription factor in the differentiation of microvillar cells or Bowman’s glands, Ascl3 deficient mice were generated by crossing *Ascl3*^*EGFP-Cre*/+^ heterozygotes, in which the entire Ascl3 coding sequence is replaced by EGFP-Cre recombinase (Fig. 1A)^29^. Homozygous Ascl3 knockout mice (*Ascl3*^*EGFP-Cre*/*EGFP-Cre*^; referred to as *Ascl3*^−/−^) were viable and survived to adulthood. There were no obvious morphological changes in the *Ascl3*^−/−^ OE (Fig. S2A), and labeling with antibodies to PLC β2, Trpm5 and AQP5 revealed that both types of microvillar cells, and Bowman’s glands were present in OE from *Ascl3*^−/−^ mice at numbers similar to wild-type controls (Fig. S2B). Thus, while Ascl3 expression is activated in a progenitor of microvillar cells and Bowman’s glands, loss of this transcription factor does not block generation or differentiation of these cell types.

### Ascl3 expression is activated in HBCs during OE regeneration

**Figure 4:**
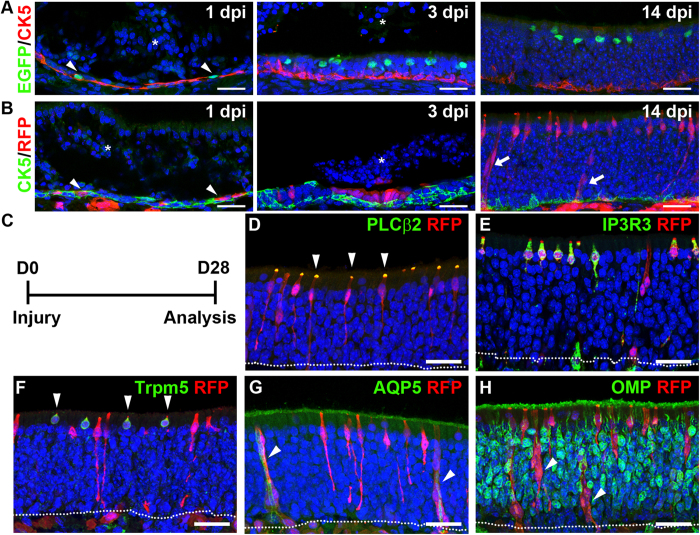
Ascl3-expressing cells regenerate microvillar cells and Bowman’s gland/ducts after injury. (**A**) Immunohistochemistry with antibodies to EGFP and CK5 on sections of OE isolated from *Ascl3*^*EGFP-Cre*/+^ after methimazole injection. At day one (1 dpi) post injury, Ascl3-expressing cells marked by EGFP co-localize with HBCs marked by CK5 expression (arrowheads). EGFP^+^ cells gradually migrate away from the basal layer toward the apical OE at 3 and 14 dpi. (**B**) Lineage tracing in *Ascl3*^*EGFP-Cre*/+^/*R26*^*tdTomato*/+^mice after injury. Antibody to RFP detected Ascl3-expressing cells co-localized with HBCs marked by CK5 antibodies at 1 dpi (arrowheads). RFP-labeled cells become apically localized at 3 and 14 dpi. White asterisk marks delaminated OE tissue. (**C)** *Ascl3*^*EGFP-Cre*/+^/*R26*^*tdTomato*/+^ mice were treated with methimazole and OE was analyzed after 28 days. (**D**–**H**) Confocal images of OE sections stained with antibodies to RFP and (**D**) PLC β2, (**E**) IP3R3, (**F**) Trpm5, (**G**) AQP5 and (**H**) OMP. RFP expression colocalized with PLC β2, IP3R3, Trpm5 and AQP5 (arrowheads). (**H**) No colocalization was detected between RFP and OMP. Arrowheads indicate Bowman’s gland ducts within the OE. Dotted line indicates basal lamina. Nuclei are stained by DAPI (blue). Scale bars: 25 μm.

The OE undergoes constant turnover under normal homeostatic conditions, and has a remarkable ability to regenerate after severe injury. Complete regeneration of the OE occurs within 2–4 weeks after chemically-induced loss of the entire epithelium. To investigate the origin of regenerated microvillar and Bowman’s gland cells in the adult OE, we used the anti-thyroid drug methimazole, which causes extensive delamination of the OE layer following intraperitoneal injection^14^,^30^. Loss of the sustentacular cells activates the quiescent HBC stem cells to regenerate all cell types in the OE^14^,^24^. *Ascl3*^*EGFP-Cre*/+^ mice (3- to 4-week-old) were given a single injection of methimazole and the OE was subsequently analyzed at specified days post-injury (dpi) (n ≥ 3 per time point). The HBCs were detected with antibody to CK5. Notably, at 1 dpi, immediately following delamination, expression of the Ascl3-EGFP was co-localized with CK5^+^ in some HBCs (Fig. 4A), revealing that Ascl3 expression is activated in a subset of HBCs early in the regeneration process. As OE regeneration proceeded, Ascl3-EGFP^+^ cells became more apically localized, and were no longer detected in the basal region (3 and 14 dpi) (Fig. 4A). To detect all cells derived from the Ascl3-positive HBCs, we used *Ascl3*^*EGFP-Cre*/+^/*R26*^*tdTomato*/+^ mice, in order to follow RFP-labeled cells derived from HBCs in which Ascl3 was expressed. Consistent with the activation of Ascl3 in some HBCs, single RFP-labeled cells were co-localized with CK5 at 1 dpi in the basal region (Fig. 4B). The RFP-labeled cells were expanded to multi-cellular clusters by 3 dpi. By 14 dpi, most RFP-labeled cells were apically located and resembled both types of microvillar cells, or exhibited the morphology of Bowman’s glands that spanned the OE (Fig. 4B, arrowheads). Double staining with antibodies to cell type-specific markers at 28 dpi confirmed that RFP label was co-localized with both PLC β2^+^/IP3R3^+^ and Trpm5^+^ microvillar cell types, as well as with AQP5^+^ Bowman’s glands (Fig. 4D–G). In contrast, mature OSNs stained with OMP antibody did not co-localize with RFP (Fig. 4H). Thus, during OE regeneration, Ascl3 expression is activated in some HBCs that serve uniquely as precursors of microvillar cells and Bowman’s glands.

### Ablation of Ascl3^+^ cells causes reduction of neuronal layer

**Figure 5:**
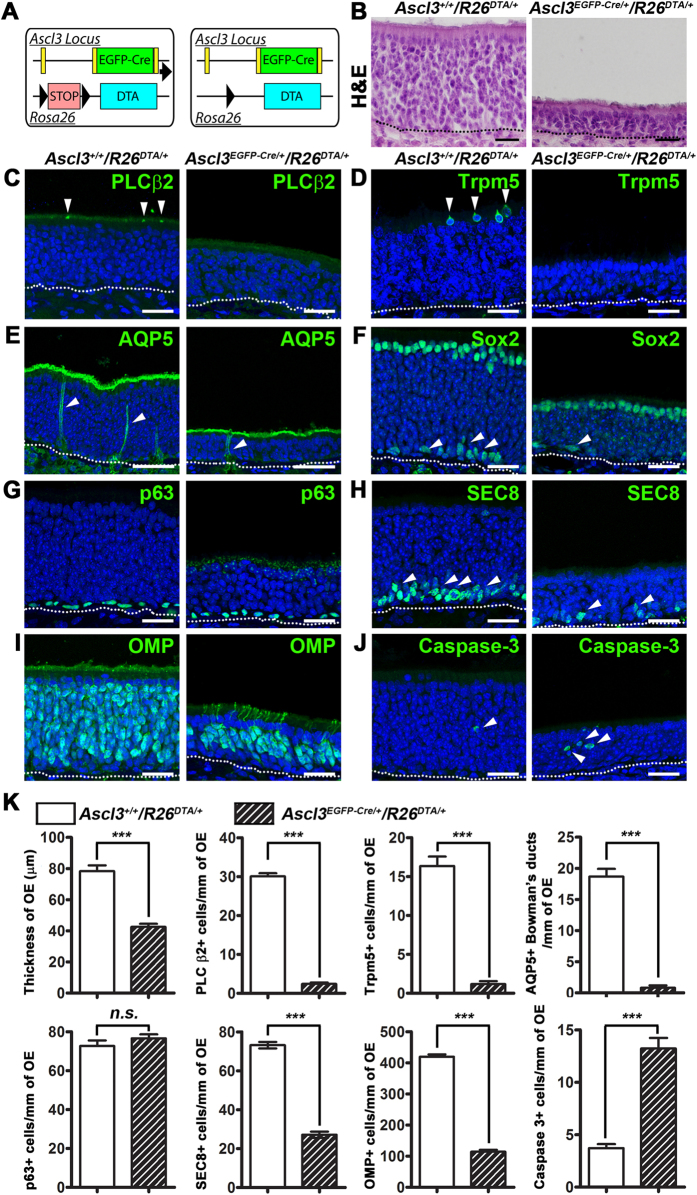
Ablation of Ascl3-expressing cells results in absence of microvillar cells and Bowman’s glands, and decreases GBCs and mature OSNs. (**A**) *Ascl3*^*EGFP-Cre/+*^*/R26*^*DTA/+*^ mice, DTA expression is activated only in the Ascl3-expressing cells. OE was isolated from *Ascl3*^+/+^/*R26*^*DTA*/+^ and *Ascl3*^*EGFP-Cre*/+^/*R26*^*DTA*/+^ mice at 2 months of age. (**B**) H&E staining showed a significant decrease in thickness of the OE in the *Ascl3*^*EGFP-Cre*/+^/*R26*^*DTA*/+^ mice compared to *Ascl3*^+/+^/*R26*^*DTA*/+^ mice. (**C**) Staining with antibody to PLC β2 (arrowheads) in OE from *Ascl3*^+/+^/*R26*^*DTA*/+^ mice and *Ascl3*^*EGFP-Cre*/+^/*R26*^*DTA*/+^mice. (**D**) Trpm5-positive microvillar cells are present at the apical surface of the OE (arrowheads) in *Ascl3*^+/+^/*R26*^*DTA*/+^ mice, but not detected in OE of *Ascl3*^*EGFP-Cre*/+^/*R26*^*DTA*/+^mice. (**E**) Antibodies to aquaporin 5 (AQP5) mark the apical surface of the duct cells in the Bowman’s glands extending through the OE in *Ascl3*^+/+^/*R26*^*DTA*/+^ mice (arrowheads). Ducts cells of the Bowman’s glands were only rarely observed in OE from *Ascl3*^*EGFP-Cre*/+^/*R26*^*DTA*/+^ mice (arrowhead). (**F**) Antibodies to Sox2 revealed no difference in number of sustentacular cells at the apical surface of the OE between mice of the two genotypes. The number of Sox2^+^ GBCs was decreased (arrowheads). (**G**) p63^+^ HBC numbers are not changed in *Ascl3*^*EGFP-Cre*/+^/*R26*^*DTA*/+^ mice. (**H**) SEC8 antibody labels GBCs near the basal layer of the OE in *Ascl3*^+/+^/*R26*^*DTA*/+^ mice (arrowheads). Significantly fewer GBCs were detected in OE of *Ascl3*^*EGFP-Cre*/+^/*R26*^*DTA*/+^mice. (**I**) Labeling with antibody to OMP showed a significant decrease in the number of labeled mature OSNs in *Ascl3*^*EGFP-Cre*/+^/*R26*^*DTA*/+^ mice compared to controls. (**J**) Antibodies to active caspase-3 showed an increase in number of apoptotic cells in the OE of *Ascl3*^*EGFP-Cre*/+^/*R26*^*DTA*/+^ mice. (**K**) Quantified results show significant decrease in the thickness of OE and the numbers of PLC β2^+^ and Trpm5^+^ microvillar cells, AQP5^+^ duct cells of the Bowman gland, but no difference in number of p63^+^ HBCs in the *Ascl3*^*EGFP-Cre*/+^/*R26*^*DTA*/+^mice. Quantification also showed a significant decrease in SEC^+^ GBCs and OMP^+^ mature OSNs in the *Ascl3*^*EGFP-Cre*/+^/*R26*^*DTA*/+^mice. In addition, an increase of caspase-3^+^ cells was observed in *Ascl3*^*EGFP-Cre*/+^/*R26*^*DTA*/+^mice (arrowheads). N ≥ 3 for *Ascl3*^+/+^/*R26*^*DTA*/+^ and *Ascl3*^*EGFP-Cre*/+^/*R26*^*DTA*/+^. ***P < 0.001. n.s., No significance. Data are shown with mean ± SEM. Dotted line indicates basal lamina. Nuclei are stained by DAPI (blue). Scale bars: (**A**), 20 μm. (**B**–**H**), 25 μm.

Both microvillar cells and Bowman’s glands are important for the maintenance of OE homeostasis^3^,^9^,^11^,^13^. Our data suggest that all microvillar cells and Bowman’s glands are derived from Ascl3-expressing progenitors. To investigate the role of these cell types in the OE, we used cell specific ablation. *Ascl3*^*EGFP-Cre*^ mice were crossed with *Rosa26*^*DTA*^, which carries a silenced diphtheria toxin A subunit (DTA) gene (Fig. 5A)^31^. Recombination by Ascl3-driven Cre will activate DTA expression and should result in ablation of both microvillar cells and Bowman’s glands in OE of the *Ascl3*^*EGFP-Cre*/+^/*Rosa26*^*DTA*/+^ (Ascl3-DTA) mice. As Ascl3 is expressed during embryonic development, and the Ascl3-Cre is constitutively active, the DTA-mediated ablation of Ascl3-expressing cells is initiated during development of the OE. We previously reported that *Ascl3*^*EGFP-Cre*/+^/*Rosa26*^*DTA*/+^ double heterozygous animals are viable, but smaller than control littermates (*Ascl3*^+/+^/*Rosa26*^*DTA*/+^)^28^. In animals 2 months old, OE was formed but displayed a significant decrease in thickness compared to control animals (distance from basal lamina to the apical surface: control, 78.35 ± 3.672; Ascl3-DTA, 42.50 ± 2.017 μm; *P* < 0.001) (Fig. 5B,K). Knockouts of CFTR, IP3R3 and NPY in microvillar cells have shown defects in the OE^9^,^11^,^13^, but the phenotype in the Ascl3-DTA mice appeared more severe. As expected, we rarely observed cells labeled with PLC β2, Trpm5 or AQP5 in OE sections from Ascl3-DTA mice, confirming the specific ablation of Ascl3 lineages (Fig. 5C–E). Quantification of PLC β2^+^ microvillar cells, Trpm5^+^ microvillar cells and AQP5^+^ Bowman’s gland ducts at the same anteroposterior level in sections from control and Ascl3-DTA OE confirmed that abundance of these cell types was significantly decreased in Ascl3-DTA mice (Fig. 5K). In contrast, no difference in the number or location of Sox2^+^ sustentacular cells and p63^+^ HBCs was observed between control and Ascl3-DTA sections (Fig. 5F,G,K). However, the number of progenitor GBCs, labeled with antibody to SEC8^23^, or antibody to Sox2, was significantly lower in the OE of Ascl3-DTA mice (Fig. 5F,H and K). The OE of Ascl3-DTA mice also had a significantly lower number of OSNs, labeled with antibody to OMP or to TuJ1 (Fig. 5I,K and Fig. S3). Finally, we examined the number of apoptotic cells using antibody to caspase-3. An increase in labeled apoptotic cells was observed and quantified in sections of Ascl3-DTA OE (at 2 months) compared to control (Fig. 5J,K). These data confirm that Ascl3^+^ progenitor cells are the exclusive source of microvillar cells and Bowman’s glands. The presence of mature OSNs labeled by OMP in the OE of Ascl3-DTA mice indicates that ablation of Ascl3-expressing cells does not block the process of neurogenesis. However, absence of microvillar cells and Bowman’s glands leads to a reduction in GBC number, as well as number of OSNs. This is correlated with an increase in apoptotic cell death. We speculate that absence of the microvillar cells and Bowman’s glands has a non-cell autonomous effect on maintenance of the OE.

### Non-neuronal cells play a role in OE regeneration

To further investigate the non-cell autonomous function of Ascl3 descendant cells, we examined OE regeneration in control and Ascl3-DTA mice using the induced injury model. Mice (3- to 4-week-old) were given a single injection of methimazole and the OE was examined after 28 days of regeneration. By 28 dpi, OE regeneration had occurred in both Ascl3 control and Ascl3-DTA mice. However, OE thickness was significantly reduced in Ascl3-DTA mice (control, 79.30 ± 1.566; Ascl3-DTA, 39.23 ± 1.383 μm; *P* < 0.001). As expected, staining with antibodies to PLC β2, Trpm5, and AQP5 at 28 dpi revealed that both types of microvillar cells and Bowman’s gland ducts were absent in the regenerated OE of Ascl3-DTA mice (Fig. S4A–C). However, sustentacular (detected with antibody to Sox2) and HBC (detected with antibody to p63) cell numbers were similar in Ascl3-DTA and control OE (Fig. S4D,E). As observed in the absence of injury, both SEC8^+^ and Sox2^+^ GBCs were significantly decreased in Ascl3-DTA mice after regeneration (Fig. S4D,F), and consistent with this, the number of OMP^+^ mature OSNs was greatly reduced (Fig. S4G). Finally, the number of active caspase-3 positive apoptotic cells was increased in the regenerated Ascl3-DTA OE (Fig. S4H). Thus, regeneration of the Ascl3-DTA OE reproduced the phenotype observed in uninjured OE of adult Ascl3-DTA mice, demonstrating that OE regeneration can occur in the absence of microvillar cells and Bowman’s glands. However, failure to develop or regenerate the complete OSN number confirms the requirement for microvillar cells, and Bowman’s glands, in promoting OE neuronal homeostasis.

**Figure 6:**
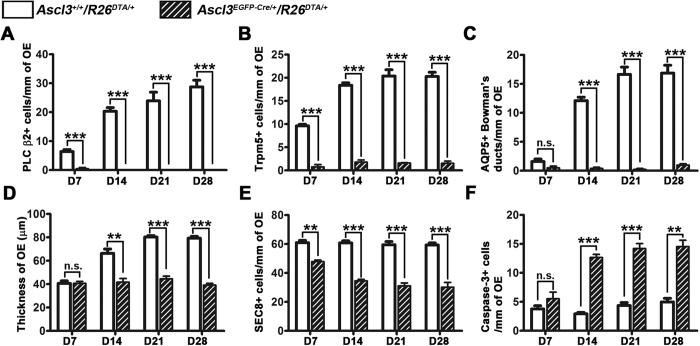
Time course of OE regeneration in the absence of non-neuronal support cells. Quantification of PLC β2^+^, Trpm5^+^ microvillar cells, duct cells of AQP5^+^ Bowman’s glands, OE thickness, SEC8^+^ GBCs and caspase-3^+^ apoptotic cells from *Ascl3*^+/+^/*R26*^*DTA*/+^ and *Ascl3*^*EGFP-Cre*/+^/*R26*^*DTA*/+^ mice at days 7, 14, 21, 28 post-injury. (**A**–**C**) Quantified results showed significantly reduced numbers of PLC β2^+^ and Trpm5^+^ microvillar cells, AQP5^+^ Bowman gland ducts in the Ascl3-DTA mice at days 7, 14, 21, 28 post-injury. (**D**) Decrease in the thickness of OE was detected from day 14 dpi during regeneration. (**E**) Decrease of SEC8^+^ GBCs was observed in the Ascl3-DTA mice at days 7, 14, 21, 28 post-injury. (**F**) More caspase-3^+^ apoptosis cells were observed in the Ascl3-DTA mice at days 7, 14, 21, 28 post-injury. N ≥ 3 for control and Ascl3-DTA mice. *P < 0.05, **P < 0.01, ***P < 0.001. n.s., No significance. Data are shown with mean ± SEM.

We compared regeneration in OE of Ascl3-control and Ascl3-DTA mice to detect the time point at which the support cells may be required. We measured OE thickness and quantified PLC β2^+^ and Trpm5^+^ microvillar cells, AQP5^+^ Bowman’s gland ducts and SEC8^+^ GBCs at 7, 14, 21, and 28 dpi. As expected, PLC β2^+^, and Trpm5^+^ microvillar cells, and AQP5^+^ Bowman’s gland ducts were nearly absent at all stages in the Ascl3-DTA (Fig. 6A–C). Notably, at 7 dpi, there was no difference in OE thickness between control and Ascl3-DTA (Fig. 6D). However, at 14 and 21 dpi, OE thickness had increased in the control but did not change in Ascl3-DTA (Fig. 6D). This suggests that neurogenesis is initiated in both, but stalls after 7 dpi in the Ascl3-DTA OE. In support of this, at 7 dpi the number of SEC8^+^ GBCs was 25% lower in the OE of Ascl3-DTA compared to that of control (Fig. 6E), and remained decreased in OE of the Ascl3-DTA at all subsequent time points (Fig. 6E). In addition, the Ascl3-DTA OE had a slightly higher number of apoptotic cells at 7 dpi, which was significantly increased over control at 14, 21, and 28 dpi (Fig. 6F). Our results indicate that maintenance of GBCs, and perhaps OSNs, is dependent on the presence of microvillar or Bowman’s gland support cells. Further investigations using the Ascl3-DTA cell ablation model could help to elucidate the critical factors or interactions generated by these cells.

## Discussion

Although specific subsets of GBCs, such as the Ascl1-positive cells, are known to be direct precursors of neuronal cells^22^, the molecular signatures which separate the other cell types in the OE are not yet clear. In this report, we identify a distinct cell lineage marked by transient expression of Ascl3 that is committed exclusively to microvillar cells and Bowman’s glands of the OE. Our data provide clear evidence that these non-neuronal cells are derived from a subpopulation of HBCs during regeneration, and cell ablation demonstrated that the source of microvillar cells and Bowman’s glands is limited to these progenitors.

Several types of microvillar cells have been identified in the OE, and are distinguished by expression of either PLC β2 and NPY, or of Trpm5^4^,^5^,^6^,^7^. Knockout of the Skn-1a (Pou2f3) transcription factor results in loss of Trpm5^+^, but not PLC β2^+^, microvillar cells^32^. Our lineage-tracing results demonstrate that all microvillar cells are derived from progenitors in which Ascl3 expression was transiently activated. Therefore, we expect that Pou2f3 must act in the lineage marked by Ascl3 expression to introduce heterogeneity of the microvillar cells. However, since the number of Trpm5^+^ cells does not decrease in Ascl3^−/−^ mice, the two factors are unlikely to act within the same pathway.

Lineage tracing studies have shown that HBCs and some GBCs can differentiate into multiple cell types, including OSNs, Bowman’s glands and sustentacular cells^14^,^21^,^22^,^33^. During development and regeneration of the OE, individual c-Kit^+^ progenitors generate microvillar cells, Bowman’s glands and OSNs, indicating that c-Kit^+^ cells are a common progenitor of neuronal and non-neuronal lineages^20^,^33^. In contrast, our results demonstrate that the Ascl3-expressing progenitors do not give rise to OSNs. These data suggest that Ascl3 is activated in a subpopulation of the c-Kit^+^ progenitor cells, and that activation of Ascl3 occurs in those cells committed to exclusively generate microvillar cells and Bowman’s glands. A recent study suggesting that Bowman’s glands and microvillar cells are separate lineages is not inconsistent with our results^33^. Our data cannot determine whether both cell types arise from a single or separate progenitors that each express Ascl3. Moreover, labeling of progenitors in that study was done relatively late after injury (7 dpi), a point at which further cell-specific commitment may have occurred.

We have previously shown that Ascl3 is expressed in the salivary gland during embryogenesis^29^. Lineage tracing demonstrated that the Ascl3-expressing cells generate both acinar and duct cells, suggesting that they are multilineage progenitors. In this report, we show that Ascl3 expression is detected in developing OE as early as E12.5, and also marks precursors of several cell types. In both tissues, Ascl3 expression in embryonic precursors appears transient. In the adult OE, Ascl3 expression is limited to PLC β2^+^ /NPY^+^ microvillar cells, and is not detected in basal progenitor or stem cells, except after injury. This is similar to our finding in the salivary glands that Ascl3 expression is limited to a subset of duct cells, which may have a specialized function^28^,^29^. Knockout of Ascl3 did not alter or block the differentiation of microvillar cells or Bowman’s glands during development or regeneration. As in the salivary gland^28^, we observed no change in the overall morphology of the OE in Ascl3 knockout mice. Although the progenitor cells in which Ascl3 is transiently activated are clearly required to generate the microvillar and Bowman’s gland cell types, the role of the Ascl3 transcription factor remains unclear.

A role for microvillar cells in regulating or maintaining the OE has been demonstrated through analysis of several gene knockouts, including CFTR^13^, NPY^9^ and IP3R3^11^. In all cases, loss of gene function in the microvillar cells affected proliferation and regenerative ability of the basal progenitor cells. It is proposed that microvillar cells coordinate signals from the external environment, and also release signals that regulate proliferation of the progenitor cells^4^,^7^,^8^,^9^,^34^. The finding that specific ablation of a cilia gene in the HBCs reduced the number of GBCs and of OSNs after chemically-induced injury supports the idea that the stem and progenitor cells may respond to non-cell autonomous signals derived from cells such as the microvillar cells^23^. Our data are consistent with this model. We show that complete ablation of both types of microvillar cells, as well as Bowman’s glands, has a striking effect on OE thickness and on the number of GBC progenitors. In contrast to ablation of the c-Kit cell population^20^, or knockout of Ascl1 in GBCs^35^, which are required for OSN generation, the ablation of Ascl3^+^ cells does not block neurogenesis. In the absence of microvillar cells and Bowman’s glands, both immature and mature neurons are still present in the uninjured and the regenerated OE. Thus, although the microvillar cells provide important signals for neuronal maintenance^9^, their absence does not impair the process of neurogenesis.

While neurogenesis does proceed, absence of the microvillar cells and Bowman’s glands leads to a deficiency in establishing or regenerating a complete OE. Our data suggest that there is no difference in regeneration up to day 7, but that proliferation and differentiation of OSNs lags at later time points. We speculate that the decrease in OE thickness, which reflects a lower number of neurons, is due to loss of signals required for stimulating or maintaining GBC cell proliferation, and preventing OSN death to establish the normal OE thickness. The decrease in GBC numbers and increase in apoptotic cells are consistent with this hypothesis.

In summary, our results demonstrate that transient Ascl3 expression marks progenitors that are strictly lineage-committed to microvillar cells and Bowman’s glands during development and regeneration. Ablation of these non-neuronal supporting cells does not block neurogenesis, but leads to impaired development or regeneration of a complete OE, and supports the idea that microvillar cells and Bowman’s glands participate in non-cell autonomous mechanisms to regulate OE integrity.

## Materials and Methods

### Animals

All mice were maintained on a C57BL/6 J background. Generation of the *Ascl3*^EGFP-Cre/+^ mice was done by replacing exon 2, including the entire coding region, with a fusion cassette encoding nuclear EGFP and Cre recombinase^29^. The *Ascl3*^EGFP-Cre/+^ mice were crossed with the *R26*^*tdTomato*^ (129S-*Gt(ROSA)26Sor*^*tm14(CAG-tdTomato)Hze*^/J) reporter strain, *R26*^*Confetti*^ (*Gt(ROSA)26Sortm1(CAG-Brainbow2.1)Cle*/J) reporter strain and the inducible cell ablation *R26*^*DTA*^ (*Gt(ROSA)26Sor*^*tm1(DTA)Jpmb*^*/J*) strain (obtained from Jackson Laboratory). Genotyping was performed with following primers: Ascl3: forward 5′-CCACCCCAGTGCCTCTACACAAAT-3′, reverse 5′-GTCGCTGGAGAAGGGCAGCAGA-3′, and Cre reverse 5′-GGTGTACGGTCAGTAAATTGGAC-3. Genotyping of *R26*^*tdTomato*^, *R26*^*Confetti*^ and *R26*^*DTA*^ mice was done with primers as recommended by supplier (Jackson Laboratory). Mice were maintained on a 12-hour light/dark cycle in a one-way, pathogen-free facility at the University of Rochester Medical Center. Food and water were provided *ad libitum*. All procedures were approved and conducted in accordance with the University of Rochester IACUC.

### Tissue preparation and staining

Embryos were taken at E12.5, E14.5, E16.5 and E18.5 and fixed in 4% paraformaldehyde at 4 °C overnight. Mice (males and females) 2 months of age were euthanized and skull bones which cover the olfactory mucosa are removed as previously described^36^. Olfactory mucosa were then fixed in 4% paraformaldehyde at 4 °C for 4 hours followed by dehydration in sucrose gradients (5–10–15%) and equilibration in 15% sucrose/OCT^TM^ compound (Sakura Finetek USA, INC) before embedding in OCT compound. Sections (10 μm) were collected on Superfrost Plus slides (Fisher Scientific) and stored at −20 °C until used for staining.

### H&E staining

Frozen sections (10 μm) were air dried and rehydrated with water. Sections were stained with hematoxylin and eosin (H&E), dehydrated through an EtOH gradient and mounted using Permount^TM^ (Fisher Scientific).

### Immunohistochemistry

Frozen sections were air dried and washed in phosphate buffer saline (PBS) to remove OCT. Sections were blocked with 5% normal donkey serum/1% BSA/0.1% Triton X-100 in PBS for 1 hour, then incubated with antibody in PBS with 1% bovine serum albumin overnight at 4 °C. Antibodies used: rabbit anti-βIII tubulin (TuJ1) (Abcam), chicken anti-GFP (Abcam), rabbit anti-Keratin-5 (BioLegend), rabbit anti-NPY (Bachem), rabbit anti-PLC β2 (Santa Cruz), rabbit anti-RFP (Rockland^TM^ antibodies & assay), goat anti-Aquaporin 5 (Santa Cruz), rabbit anti-caspase-3 (Abcam), mouse anti-IP3R3 (BD Bioscience), goat anti-OMP (Wako Chemicals), mouse anti-p63 (Santa Cruz), mouse anti-SEC8 (BD Bioscience), rabbit anti-Sox2 (EMD Millipore) and rabbit anti-Trpm5 (Alomone Labs). The detailed primary antibody information is listed in supplementary Table 1. Sections were heated in 0.1 M sodium citrate for 10 minutes for antigen retrieval. After cooling, sections were blocked with 5% normal donkey serum/1% BSA/0.1% Triton X-100 in PBS for 1 hour, then incubated with antibody in PBS with 1% bovine serum albumin overnight at 4 °C. Cy2- or Cy3-conjugated secondary antibodies (1:500, Jackson ImmunoResearch Laboratories) and Alexa Flour 488-conjugated anti-chicken antibody (1:100, Invitrogen) were used. Tyramide signal amplification kit (Invitrogen) was used to visualize IP3R3, p63 and Sox2 signal according to manufacturer’s protocol. Nuclei were stained with DAPI (Invitrogen). Sections were mounted in IMMUNE-MOUNT (Thermal Scientific). Fluorescent images were acquired on Leica TCS SP5 system using 40x oil immersion objective with a zoom of 1, 1.5 or 2 and processed using Adobe Photoshop. Figures were assembled using Adobe Photoshop.

### *In situ* hybridization

Ascl3 sense and antisense riboprobes were described previously^29^ and generated using the digoxigenin-labeling kit (Roche) followed by incubation with Anti-Digoxigenin-AP (Roche). The signal was detected using BM Purple (Roche). All frozen sections of mouse olfactory epithelium used were 10 μm. Images were taken using an Olympus DX41 microscope with a DP71 camera, analyzed on DP-BSW-V3.2 software and processed using Adobe Photoshop (Olympus America Inc). Figures were assembled using Adobe Photoshop.

### Olfactory epithelial lesion

Methimazole (U.S. Pharmacopeial) (50 mg/kg of body weight) in saline was intraperitoneally injected into 3–4 week-old mice (both male and female). Olfactory mucosa was isolated at 1, 3, 7, 14, 21 and 28 days after injury and processed as described above. N ≥ 3 mice of each genotype were analyzed for each time point.

### Quantitative analysis

Thickness of the olfactory epithelium was measured in mice of different genotypes along the septum from 3–6 images at the same anteroposterior level by Image J (NIH) software. To analyze different cell types, 3–6 images were taken from dorsal-medial, ventral medial, dorsal lateral and ventral lateral regions at the same anteroposterior level. ImageJ software was used to measure the length along the basal surface of the OE from each image (in micrometers). Cell numbers were counted from blindly assigned images of each genotype. Data are shown as mean ± SEM per mm of OE. N ≥ 3 mice of each genotype were analyzed. Results were analyzed using the unpaired student’s t-test with GraphPad Prism 5 (GraphPad Software). Statistical significance was set at *P* < 0.05.

## Additional Information

**How to cite this article**: Weng, P.-L. *et al*. Ascl3 transcription factor marks a distinct progenitor lineage for non-neuronal support cells in the olfactory epithelium. *Sci. Rep.* **6**, 38199; doi: 10.1038/srep38199 (2016).

**Publisher's note:** Springer Nature remains neutral with regard to jurisdictional claims in published maps and institutional affiliations.

## Supplementary Material

Supplementary Information
