# Supplementary material for: Ascl3 transcription factor marks a distinct progenitor lineage for non-neuronal support cells in the olfactory epithelium
Source: Sci Rep. 2016 Dec 2;6:38199. doi: 10.1038/srep38199 (PMC5133605; doi:10.1038/srep38199)
Supplement: Supplementary Information [file srep38199-s1.pdf]

Ascl3 transcription factor marks a distinct progenitor lineage for non-neuronal support cells in the olfactory epithelium

Pei-Lun Weng, Mridula Vinjamuri & Catherine E. Ovitt*

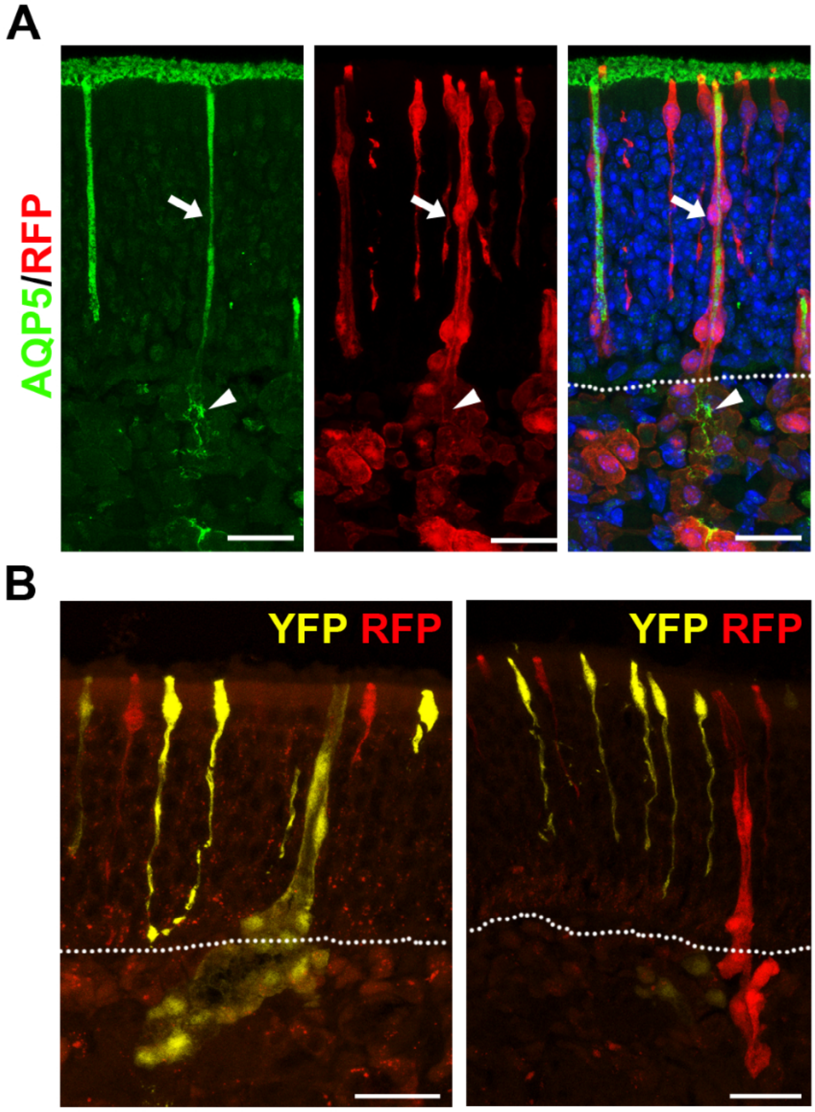

Supplementary Figure 1. Ascl3-expressing cells are precursors for microvillar cells and Bowman's glands (A) OE harvested from *Ascl3*$^{EGFP\text{-}Cre/+}$ */R26*$^{tdTomato/+}$ mice at 2 months of age. Immunohistochemistry was performed using antibodies to tdTomato (RFP) and Bowman's gland marker AQP5. RFP expression colocalized with AQP5, which labels the duct cells spanning the OE (arrow) and gland cells underneath the basal lamina (arrowhead). **(B)** OE harvested from *Ascl3*$^{EGFP\text{-}Cre/+}$ */R26*$^{Confetti/+}$ mice at 2 months of age. YFP and RFP labeled Bowman's glands and microvillar cells. Dotted line indicates basal lamina. Nuclei are stained by DAPI (blue). Scale bars: 25μm.

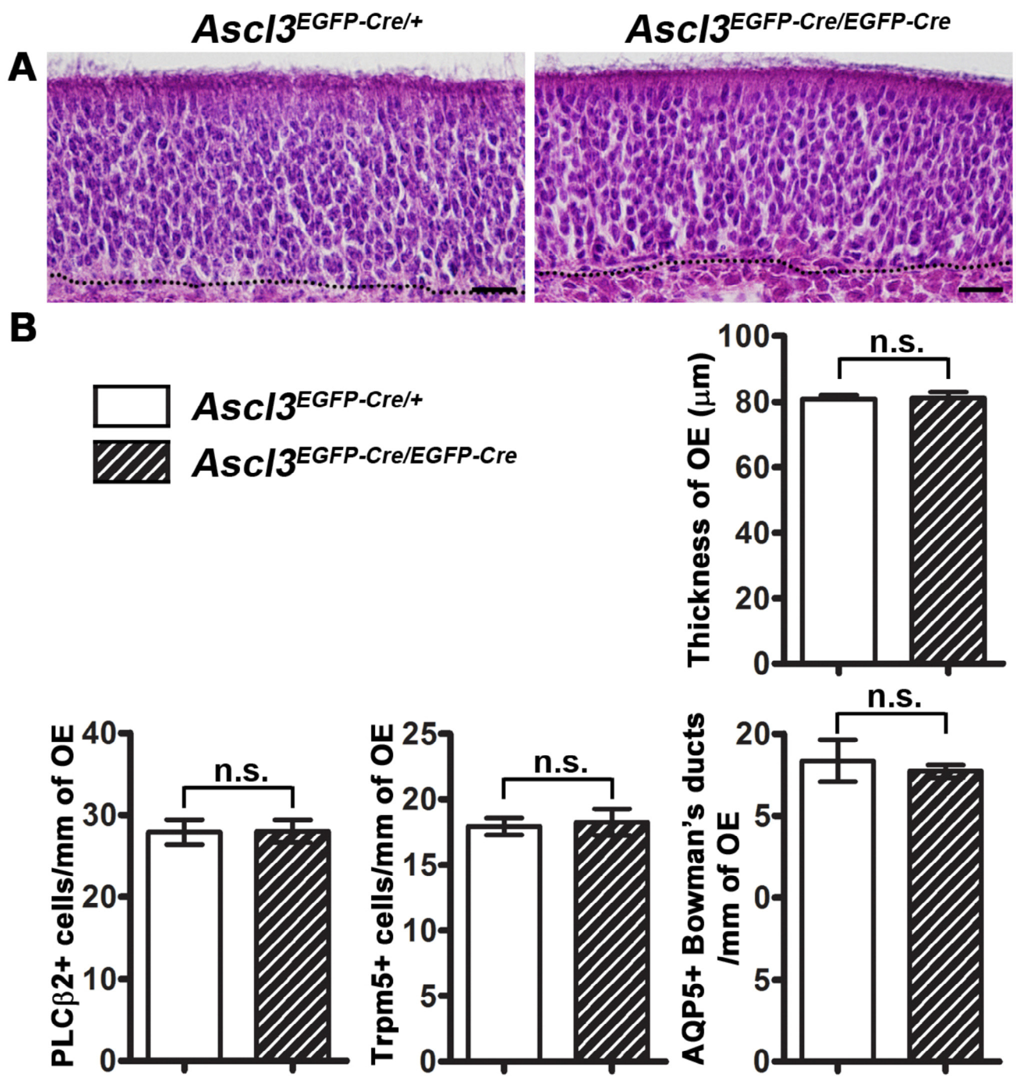

Supplementary Figure 2. Knockout of Ascl3 transcription factor does not affect olfactory epithelium thickness or differentiation of microvillar cells or Bowman's glands. OE isolated from *Ascl3*$^{EGFP\text{-}Cre/+}$ and *Ascl3*$^{EGFP\text{-}Cre/\ EGFP\text{-}Cre}$ mice at 2 months of age. (A) H&E staining showed no difference in thickness of the OE in the *Ascl3*$^{EGFP\text{-}Cre/\ EGFP\text{-}Cre}$ mice in comparison to OE from *Ascl3*$^{EGFP\text{-}Cre/+}$ mice. (B-D) Quantification showed that knockout of Ascl3 caused no changes in the OE thickness, the number of PLCβ2^{+}, or Trpm5^{+} microvillar cells, or in the AQP5^{+} duct cells of Bowman glands. N = 3 for *Ascl3*$^{EGFP\text{-}Cre/+}$ and *Ascl3*$^{EGFP\text{-}Cre/\ EGFP\text{-}Cre}$ mice. n.s., No significance. Data are shown with mean ± SEM. Dotted line indicates basal lamina. Scale bars: 20μm.

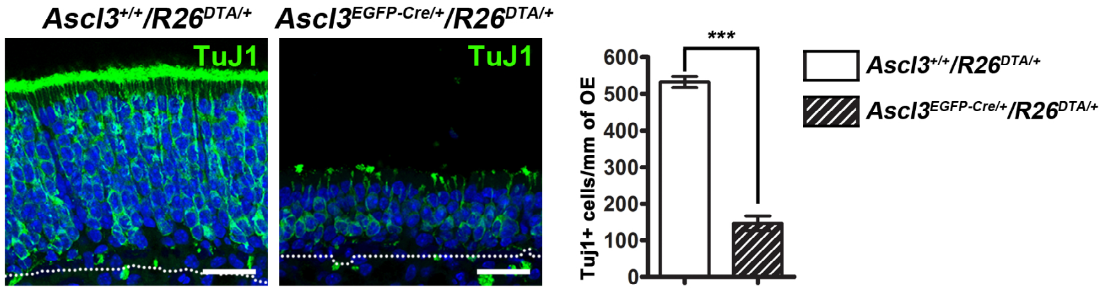

Supplementary Figure 3. Ablation of Ascl3-expressing cells results in decreased number of OSNs. Labeling with antibody to TuJ1 showed a lower number of labeled OSNs in *Ascl3*$^{EGFP\text{-}Cre/+}$/*R26*$^{DTA/+}$ mice compared to controls. Quantified results measured a significant decrease in TuJ1^{+} OSNs in the *Ascl3*$^{EGFP\text{-}Cre/+}$/*R26*$^{DTA/+}$ mice. N ≥ 3 for *Ascl3*$^{+/+}$/*R26*$^{DTA/+}$ and *Ascl3*$^{EGFP\text{-}Cre/+}$/*R26*$^{DTA/+}$. *** P <0.001. Data are shown with mean ± SEM. Dotted line indicates basal lamina. Nuclei are stained by DAPI (blue). Scale bars: 20μm.

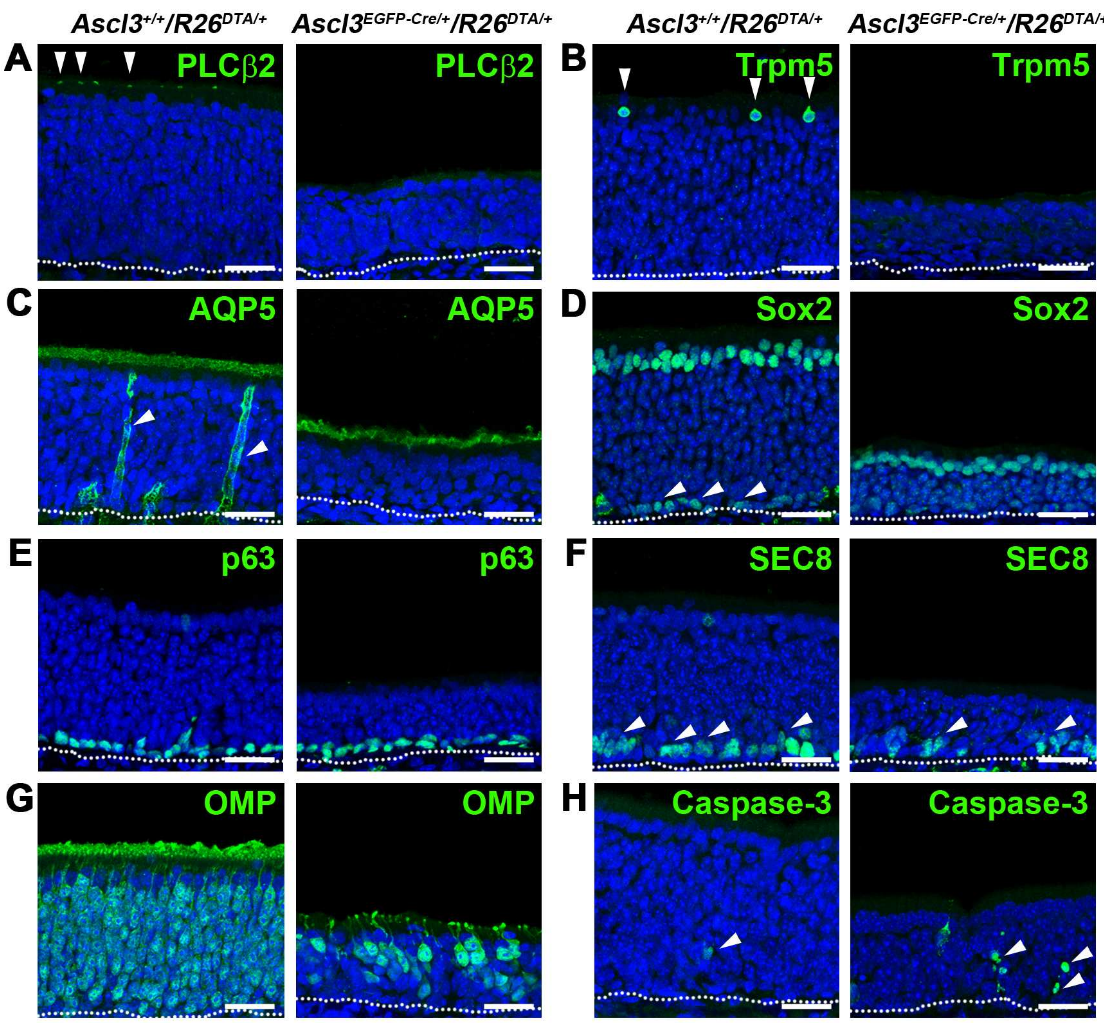

Supplementary Figure 4. OE regeneration does not require non-neuronal support cells derived from Ascl3 lineage. OE isolated from *Ascl3*$^{+/+}$/*R26*$^{DTA/+}$ and *Ascl3*$^{EGFP\text{-}Cre/+}$/*R26*$^{DTA/+}$ mice at day 28 post-treatment (28 dpi) with methimazole. (A) Staining with antibody to PLC β2 detected microvillar cells (arrowheads) in OE from *Ascl3*$^{+/+}$/*R26*$^{DTA/+}$ mice, which were absent in OE of *Ascl3*$^{EGFP\text{-}Cre/+}$/*R26*$^{DTA/+}$mice. (B) Trpm5 antibody detected microvillar cells at the most apical surface of the OE (arrowheads) in *Ascl3*$^{+/+}$/*R26*$^{DTA/+}$ mice, but very few were detected in OE of *Ascl3*$^{EGFP\text{-}Cre/+}$/*R26*$^{DTA/+}$mice. (C) Antibody to AQP5 marked duct cells of the Bowman's glands spanning the OE in *Ascl3*$^{+/+}$/*R26*$^{DTA/+}$ mice (arrowheads). Bowman's gland ducts were only rarely detected in *Ascl3*$^{EGFP\text{-}Cre/+}$/*R26*$^{DTA/+}$ mice. (D) Staining with antibody to Sox2 showed no difference in number of sustentacular cells

in the OE between mice of the two genotypes. However, the number of Sox2+ GBCs was decreased (arrowheads). (E) Antibody to p63^{+} detected no change in number of HBCs in *Ascl3*$^{EGFP\text{-}Cre/+}$/*R26*$^{DTA/+}$ mice. (F) SEC8 antibody labeled GBCs near the basal layer of the OE in *Ascl3*$^{+/+}$/*R26*$^{DTA/+}$ mice (arrowheads). Significantly fewer GBCs were detected in OE of *Ascl3*$^{EGFP\text{-}Cre/+}$/*R26*$^{DTA/+}$mice. (G) Labeling with antibody to OMP showed a significant decrease in mature OSN number in *Ascl3*$^{EGFP\text{-}Cre/+}$/*R26*$^{DTA/+}$ compared to *Ascl3*$^{+/+}$/*R26*$^{DTA/+}$. (H) Antibody to caspase-3 showed increased number of apoptotic cells in *Ascl3*$^{EGFP\text{-}Cre/+}$/*R26*$^{DTA/+}$ mice (arrowheads). Dotted line indicates basal lamina. Nuclei are stained by DAPI (blue). Scale bars: (A), 20μm. (B-H), 25μm.

Supplementary Table. 1 List of Antibodies

Primary antibody	Dilution	Source/Catalog number	Cell type(s) marked	Proof of specificity in references
Goat α-AQP5	1:100	Santa Cruz/sc-9890	Bowman's gland/duct	Chai, R.C. *et al.* (2013) 1
Rabbi α-Keratin-5 (CK5)	1:200	Biolegend/PRB-160P	HBCs	Maurya, D.K. *et al.* (2015) 2
Chicken α-GFP	1:500	Abcam/ab13970	GFP	Chen, M. *et al.* (2014) 3
Mouse α-IP3R3	1:200	BD Bioscience/610312	Microvillar cells	Pfister, S. *et al.* (2012) 4
Rabbit α-βIII tubulin (TuJ1)	1:100	Abcam/ab18207	Olfactory sensory neurons	Fletcher, Russell B. *et al.* (2011) 5
Rabbit α-NPY	1:500	Bachem/T-4070	Microvillar cells	Jia, C. & Hegg, C.C. (2010) 6
Mouse α-p63	1:200	Santa Cruz/sc-8431	HBCs	Packard, A. *et al.* (2011) 7
Rabbit α-PLC β2	1:100	Santa Cruz/sc-206	Microvillar cells	Pfister, S. *et al.* (2012) 4
Rabbit α-RFP	1:500	Rockland™ antibodies &assay/600-41-379	RFP	Aure, M.H. *et al.* (2015) 8
Mouse α-SEC8	1:250	BD Bioscience/610658	GBCs	Joiner, A.M. *et al.* (2015) 9
Rabbit α-Sox2	1:100	EMD Millipore/AB5603	Sustentacular cells	Kam, J.W. *et al.* (2016) 10
Rabbit α-Trpm5	1:500	Alomone Labs/ACC-045	Microvillar cells	Yamaguchi, T. *et al.* (2014) 11
Goat α-OMP	1:1000	WAKO/544-1001-WAKO	Mature olfactory sensory neurons	Packard, A. *et al.* (2011) 7
Rabbit α-active Caspase-3	1:100	Abcam/ab2302	Apoptosis cells	Finelli, M.J. *et al.* (2013) 12

References:

1. Chai, R.C. *et al.* AQP5 is differentially regulated in astrocytes during metabolic and traumatic injuries. *Glia* **61**, 1748-65 (2013).
2. Maurya, D.K. *et al.* Development of the Olfactory Epithelium and Nasal Glands in TMEM16A-/- and TMEM16A+/+ Mice. *PLoS One* **10**, e0129171 (2015).
3. Chen, M. *et al.* Wnt-responsive Lgr5(+) globose basal cells function as multipotent olfactory epithelium progenitor cells. *J Neurosci* **34**, 8268-76 (2014).
4. Pfister, S. *et al.* Characterization and turnover of CD73/IP(3)R3-positive microvillar cells in the adult mouse olfactory epithelium. *Chem Senses* **37**, 859-68 (2012).
5. Fletcher, Russell B. *et al.* p63 Regulates Olfactory Stem Cell Self-Renewal and Differentiation. *Neuron* **72**, 748-759 (2011).
6. Jia, C. & Hegg, C.C. NPY mediates ATP-induced neuroproliferation in adult mouse olfactory epithelium. *Neurobiol Dis* **38**, 405-13 (2010).
7. Packard, A., Schnittke, N., Romano, R.A., Sinha, S. & Schwob, J.E. DeltaNp63 regulates stem cell dynamics in the mammalian olfactory epithelium. *J Neurosci* **31**, 8748-59 (2011).
8. Aure, M.H., Konieczny, S.F. & Ovitt, C.E. Salivary gland homeostasis is maintained through acinar cell self-duplication. *Dev Cell* **33**, 231-7 (2015).
9. Joiner, A.M. *et al.* Primary Cilia on Horizontal Basal Cells Regulate Regeneration of the Olfactory Epithelium. *J Neurosci* **35**, 13761-72 (2015).
10. Kam, J.W. *et al.* RGMB and neogenin control cell differentiation in the developing olfactory epithelium. *Development* **143**, 1534-46 (2016).
11. Yamaguchi, T. *et al.* Skn-1a/Pou2f3 is required for the generation of Trpm5-expressing microvillous cells in the mouse main olfactory epithelium. *BMC Neurosci* **15**, 13 (2014).
12. Finelli, M.J., Murphy, K.J., Chen, L. & Zou, H. Differential phosphorylation of Smad1 integrates BMP and neurotrophin pathways through Erk/Dusp in axon development. *Cell Rep* **3**, 1592-606 (2013).
